# Tumor Cell-derived Extracellular Vesicles in Modulating Phenotypes and Immune Functions of Macrophages: Mechanisms and Therapeutic Applications

**DOI:** 10.7150/jca.84632

**Published:** 2023-05-08

**Authors:** Jia-Wen Tian, He-Jing Zhang, Si-Yuan Li, Yong-Lin Guo, Gang Chen, Zi-Li Yu

**Affiliations:** 1 The State Key Laboratory Breeding Base of Basic Science of Stomatology (Hubei-MOST) & Key Laboratory of Oral Biomedicine Ministry of Education, School and Hospital of Stomatology, Wuhan University, Wuhan 430079, China.; 2 Department of Oral and Maxillofacial Surgery, School and Hospital of Stomatology, Wuhan University, Wuhan 430079, China.; 3 TaiKang Center for Life and Medical Sciences, Wuhan University, Wuhan 430071, China.; 4 Frontier Science Center for Immunology and Metabolism, Wuhan University, Wuhan 430071, China.

**Keywords:** Extracellular vesicles, tumor cell, macrophage, intercellular communication, therapeutic application

## Abstract

Tumor tissues consist of tumor cells and tumor stroma, which is structured by non-tumor cells and the extracellular matrix. Macrophages are the predominant immune cells in the tumor microenvironment (TME). Based on the intimate interaction between macrophages and tumor cells, macrophages are closely involved in tumor initiation and progression, playing a key role in tumor formation, angiogenesis, metastasis, and immune escape. Extracellular vesicles (EVs) are a group of membrane-enclosed structures secreted by almost all cell types. As crucial mediators of cell-to-cell communication, EVs play a role in various physiological processes and the development of diseases including cancer. According to numerous studies, tumor cell-derived extracellular vesicles (T-EVs) could highly modulate the phenotypes and functions of macrophages, thus promoting tumor development. Herein, we comprehensively introduce the role of T-EVs in regulating the M1/M2 phenotypes and immune functions of macrophages, including cytokine secretion, expression of immune regulatory molecules on the membrane, phagocytosis, and antigen presentation. More importantly, based on the regulatory effects of T-EVs on macrophages, we propose several potential therapeutic approaches that may guide future attempts to increase the effectiveness of cancer therapy.

## Introduction

Tumor tissue consists of tumor cells and tumor stroma. Tumor stroma contains the extracellular matrix and various non-tumor cells, including fibroblasts, endothelial cells, and immune cells (e.g., macrophages and T cells), forming a complex tumor microenvironment (TME) 1, 2. In the TME, tumor cells and stromal cells interact in various ways, including direct cell-to-cell contact, the production of soluble protein-based factors, and the release of extracellular vesicles (EVs) 3. This intercellular communication plays a key role in tumor growth, invasion, and metastasis 1.

**Figure 1:**
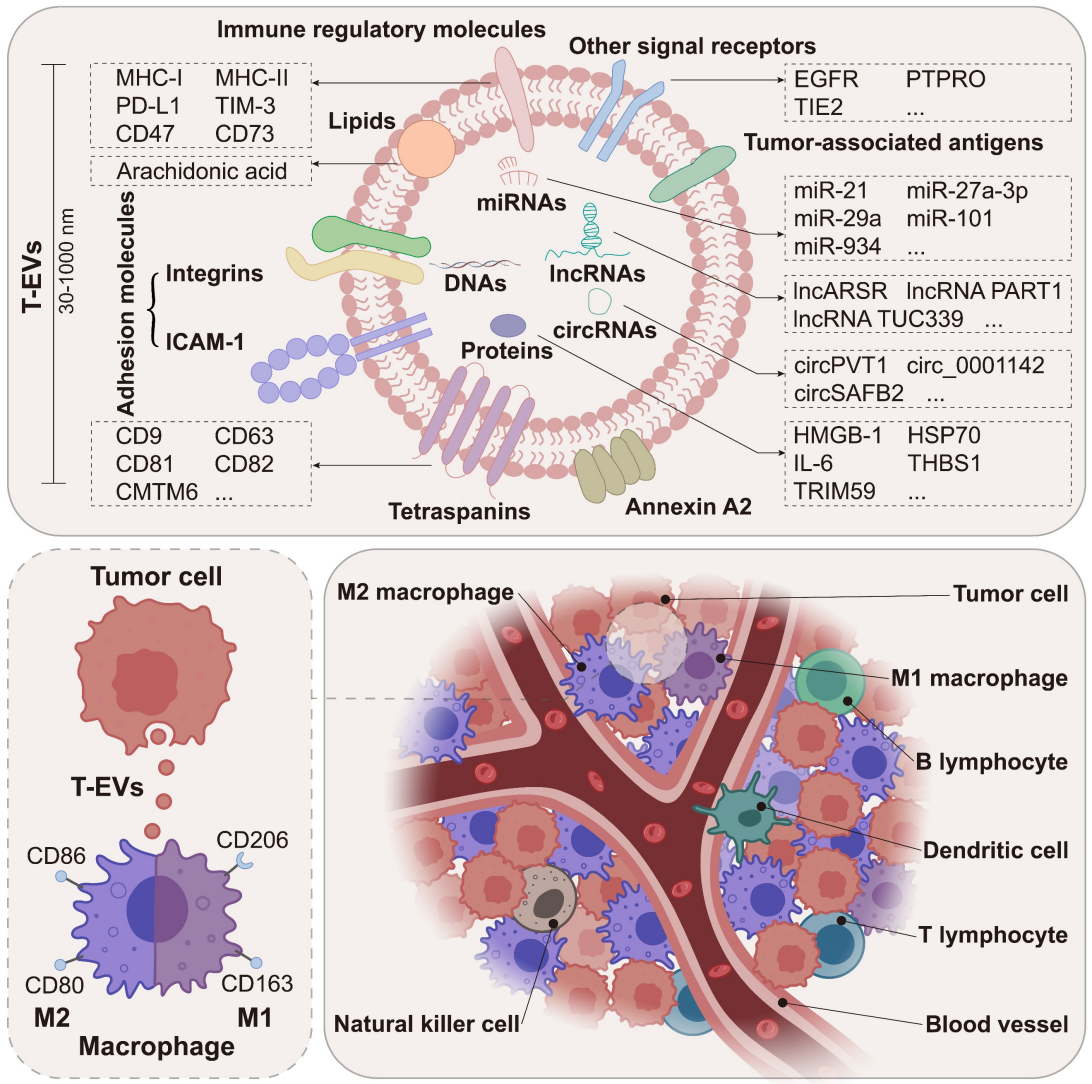
T-EVs deliver multiple cargos to macrophages within the TME. Macrophages are the predominant immune cells in the TME. T-EVs deliver multiple cargos to macrophages, including DNAs, RNAs, proteins, and lipids. Macrophages are broadly classified into M1 and M2 subtypes.

As the predominant immune cells within the TME 4-6, through the intimate and extensive interactions with tumor cells, macrophages are closely involved in tumor initiation and progression, playing a key role in tumor formation, angiogenesis, metastasis, and immune escape 7. Macrophages in the TME are mainly recruited from circulating monocytes and are often referred to as tumor-associated macrophages (TAMs). Notably, macrophages are highly plastic and can change their phenotypes and functions in response to environmental stimuli. EVs are a group of membrane-enclosed structures secreted by almost all types of cells 8. As crucial mediators of cell-to-cell communication, EVs play a role in various physiological processes and the development of diseases, including cancer 9, 10. According to numerous studies, tumor cell-derived EVs (T-EVs) could effectively modulate the phenotypes and functions of macrophages through multiple cargos, thus promoting tumor development (Figure 1). Targeting the interactions between tumor cells and macrophages has been a hot area of research in cancer therapy.

Herein, we comprehensively introduce the role of T-EVs in regulating the phenotypes (M1/M2) and immune functions of macrophages, including cytokine secretion, expression of immune regulatory molecules on the membrane, phagocytosis, and antigen presentation. Most importantly, given the critical regulatory effects of T-EVs on macrophages and the TME, there is an urgent need for the suppression or elimination of T-EVs to improve the therapy and prognosis of cancer patients. Herein, we propose several potential therapeutic approaches that may guide future attempts to increase the effectiveness of cancer therapy.

## Biogenesis and secretion of T-EVs

EVs are a diverse population of membrane vesicles generated via multiple mechanisms. Based on their formation mechanism and size, EVs are generally categorized as exosomes and microvesicles (MVs). Exosomes (30-150 nm in diameter) originate from the endosomal pathway that involves sorted endosomes and intracellular multivesicular bodies (MVBs) containing intraluminal vesicles (ILVs) 9. Due to the fusion of MVBs and the plasma membrane, ILVs could be released into the pericellular space. These ILVs may represent pre-secreted exosomes, but the majority of ILVs may not be released. The process of producing exosomes depends on the endosomal sorting complex required for transport (ESCRT) complexes, which aid in clustering cargos, creating membrane buds, storing cargo within the buds, and facilitating scission 11. According to research, a number of these key regulators are overexpressed and/or hyperactivated in a wide variety of tumor cells, accounting for the high production of T-EVs 12-14. MVs (100-1000 nm in diameter and larger than exosomes) are produced by direct plasma membrane budding 15, 16.

EVs inherit biomolecules (e.g., nucleic acids, proteins, and lipids) from their donor cells, and the level of EV cargos is regulated by a variety of molecules in their donor cells 17. A study conducted by Chen et al., for example, indicated that knockdown of the ESCRT subunit HRS in malignant melanoma cells led to downregulated PD-L1 level in EVs, suggesting that HRS may be an important target in cargo sorting of EVs 18. EVs deliver their cargos to recipient cells, contributing to intercellular communication 17. It is commonly acknowledged that tumor cells generate more EVs than non-tumor cells, and both cell-intrinsic and external signals may influence the EV release of tumor cells 19. Dysregulation of T-EV cargos and T-EV release has a substantial impact on the interactions between tumor cells and macrophages in the TME, as well as in metastatic and premetastatic niches.

## Tumor cell-derived EVs affect phenotypes of macrophages

**Figure 2:**
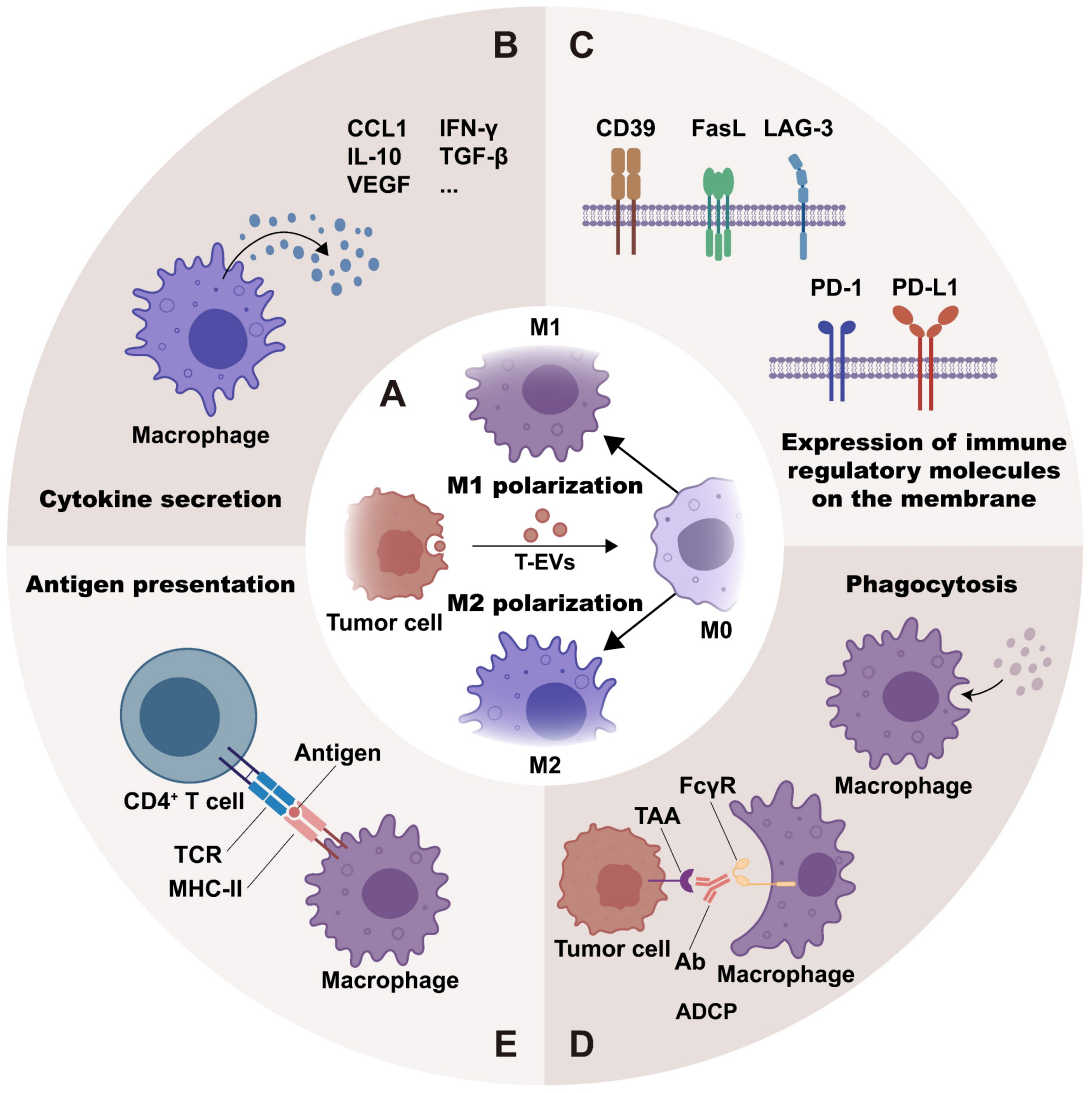
T-EVs regulate macrophage phenotypes and immune functions: T-EVs may mediate macrophage polarization toward the M1 or M2 phenotype (**A**) through their contents and surface molecules. Moreover, T-EVs are effective in regulating macrophage immune functions. They do this by regulating cytokine secretion (**B**), expression of immune regulatory molecules on the membrane (**C**), phagocytosis (**D**), and antigen presentation (**E**) of macrophages.

Regulated by the stimuli from the milieu, macrophages are classified into different phenotypes according to their functional orientation. The M1 phenotype and the M2 phenotype are the two major polarization states of macrophages 20. It is generally recognized that M1 macrophages mediate anti-tumor activity, whereas M2 macrophages are linked with pro-tumorigenic functions, including promoting tumor growth, metastasis, and angiogenesis 21. Research suggests that RNAs, proteins, and lipids in T-EVs may help polarize macrophages, mostly toward the M2 phenotype (Figure 2A and Table S1).

### T-EV-RNAs affect phenotypes of macrophages

RNAs, including microRNAs (miRNAs), long non-coding RNAs (lncRNAs), and circular RNAs (circRNAs), have different effects on cell growth, proliferation, and metabolism through different pathways 22. T-EV-RNAs may alter macrophage polarization through the activation of signaling pathways, signal transduction, transcriptional regulation, and post-transcriptional regulation 23. Specifically, miRNAs silence genes by directly targeting mRNAs, while lncRNAs and circRNAs sponge with miRNAs to indirectly affect protein expression 23.

#### miRNAs

miRNAs are the most abundant kind of RNAs encapsulated in EVs 24. Under the protection of EV membrane structure, miRNAs can be stably expressed in the extracellular space 25. Valadi et al. found evidence that miRNAs could be functionally transported from donor cells to recipient cells by EVs, which would be a key part of EV-mediated intercellular communication 26. T-EV-encapsulated miRNAs may regulate the phenotypes of macrophages, thus influencing tumor development and formation. In most cases, T-EVs induce macrophages to an M2 phenotype, promoting tumor development. For instance, EVs produced by colorectal cancer (CRC) cells carried miR-934 into macrophages, suppressed PTEN expression and activated the PI3K/AKT signaling pathway to promote M2 macrophage polarization 27. Interestingly, by secreting CXCL13, polarized M2 macrophages enhanced CRC liver metastasis by triggering a positive feedback loop involving CXCL13, CXCR5, NF-κB, p65, and miR-934 in CRC 27.

Hypoxia is a common characteristic of solid tumors. Hypoxia may increase the production of T-EVs that carry miRNAs, and the miRNAs encapsulated in T-EVs may cause M2-like macrophage polarization. Chen et al. demonstrated that hypoxia increased the level of miR-940 in EVs produced by epithelial ovarian cancer (EOC) cells, and that EV-miR-940 delivered to macrophages encouraged M2-phenotype polarization, hence promoting EOC proliferation and migration 28. Wang et al. discovered that miR-301a had a high expression in EVs released by hypoxia-treated pancreatic cancer cells and could be carried to macrophages 29. EV-miR-301a induced macrophage M2 polarization in a HIF-1a or HIF-2-dependent way and subsequently facilitated the invasion, migration, and epithelial-mesenchymal transition (EMT) of pancreatic cancer cells 29. Qian et al. suggested that hypoxic glioma-derived EVs, in comparison to normoxic glioma-derived EVs, markedly triggered M2 macrophage polarization, which promoted the establishment of the immunosuppressive microenvironment 30.

In addition to the pro-tumoral M2 phenotype, specific miRNAs (e.g., miR-9, miR-33, miR-125b, and miR-130) in T-EVs may polarize macrophages towards the anti-tumor M1 phenotype 31-33. Tong et al. reported that miR-9 was abundant in HPV^+^ head and neck squamous cell carcinoma (HNSCC) cell-derived EVs and could be carried to macrophages and promote M1 polarization by downregulating PPAR, hence promoting HNSCC radiosensitivity 31. Moradi-Chaleshtori et al. revealed that miR-130 and miR-33 overexpression in EVs of human breast cancer cells inhibited tumor development by reprogramming macrophages from the M2 to the M1 phenotype 32. This reprogramming process could be used as a therapeutic method for tumor 32.

#### Other RNAs

lncRNAs are RNAs that have a length of over 200 nucleotides and have a restricted potential to encode proteins. There is mounting evidence that lncRNAs have a role in a wide range of biological processes, from development and physiology to pathology, including tumorigenesis and tumor progression 34. According to a study by Cao et al., lncRNA-MM2P expression was upregulated during M2 polarization but downregulated during M1 polarization, indicating that lncRNA may influence macrophage polarization 35. Higher amounts of lncRNA TUC339 were found in EVs of hepatocellular carcinoma (HCC) cells, and these EVs could be taken up by macrophages and polarize macrophages to the M2 phenotype 36. Liang et al. revealed that EVs produced by CRC cells carried lncRNA-RPPH1 into macrophages and induced macrophage M2 polarization, increasing CRC cell proliferation and metastasis 37.

circRNAs are a type of non-coding RNA that is generated through a process known as back-splicing, which involves the circularization of exons or introns. Studies have shown that circRNAs could be packaged into EVs and play a key role in the development of tumors. Chen et al. reported that circFARSA in EVs of non-small cell lung cancer cells induced M2 macrophage polarization via PTEN ubiquitination and degradation, which promoted tumor metastasis 38. Pan et al. recently reported that glioma cell-derived EVs transmitted circNEIL3 to infiltrated TAMs, thereby promoting macrophage polarization toward the immunosuppressive phenotype characterized by CD11b and CD163 expression by stabilizing IGF2BP3 protein, which promoted glioma progression 39. Besides, EVs derived from esophageal squamous cell carcinoma (ESCC) cells could transport hsa-circ-0048117 to macrophages and mediate M2 polarization as a miR-140 sponge 40.

### T-EV-proteins affect phenotypes of macrophages

Studies have shown that T-EVs may act as trafficking vehicles to transport proteins from tumor cells to macrophages, modulating macrophage polarization. Among these proteins are immune checkpoint molecules and other proteins.

#### Immune checkpoint molecules

The term "immune checkpoints" describes a group of ligands present on the surface (membrane) of antigen-presenting cells that bind to specific receptor partners on T cells 41. This interaction can result in the activation and differentiation of T cells (stimulatory checkpoints) or the suppression of the immune response (inhibitory checkpoints) 41. Immune checkpoint molecules serve as immune regulators and are critical for maintaining self-tolerance 41. Whereas tumor cells may express high levels of inhibitory checkpoints that inhibit normal anti-tumor immune responses, resulting in immune escape 42.

T-EVs may inherit inhibitory checkpoint molecules from tumor cells and thus limit T-cell activation as well as polarize macrophages towards pro-tumoral M2 phenotype 18, 43, 44. Chen et al. revealed that metastatic melanoma cells generated EVs with PD-L1, which inhibited CD8^+^ T cells and promoted tumor growth 18. Interestingly, Li et al. discovered that TIM-3 shuttled by human melanoma cell-derived EVs could inhibit the immune function of CD4^+^ T cells and induce macrophage M2 polarization, which ultimately promoted melanoma cell growth and metastasis 45. According to current studies, T-EVs could transport immune checkpoint molecules (e.g., PD-L1 and TIM-3) to macrophages and induce M2 macrophage polarization. Liu et al. found that xCT inhibitor-treated melanoma cells released EVs with a high expression level of PD-L1, which could upregulate macrophage PD-L1 expression and lead to M2 macrophage polarization 43. Similarly, Cheng et al. found that EVs of osteosarcoma cells induced macrophage M2 polarization through Tim-3, promoting tumor metastasis, invasion, and EMT of osteosarcoma 44.

The blocking of immune checkpoints is an essential component of immunotherapy, the significance of which in treating cancer is well recognized. Ideally, immune checkpoint blockade enables efficient immune responses, and in the meanwhile, maintains sufficient self-tolerance and prevents excessive immune cell activation 46. Given that immune checkpoint blockade not only rescues T cells from immunosuppression and reduces resistance to immunotherapy, but also modifies macrophage phenotypes and substantially affects therapeutic outcomes, immune checkpoint blockade could be regarded as a highly effective cancer treatment method.

#### Other proteins

In addition to immune checkpoint molecules, studies have identified other T-EV proteins that trigger M1 or M2 macrophage polarization. TEK gene-encoded tyrosine kinase with immunoglobulin and epidermal growth factor homology domains 2 (TIE2) is the receptor for angiopoietins and performs essential functions in vascular remodeling during inflammation and tumor angiogenesis 47, 48. TIE2-expressing macrophages (TEMs) are a group of TAMs with characteristics of pro-tumorigenic phenotypes, supporting tumor angiogenesis and being related to cervical cancer progression. The direct transfer of TIE2 protein to macrophages through EVs led to IL-10 upregulation and TNF downregulation in TEMs, which were consistent with M2 macrophages 49. Dong et al. showed that EV-protein tyrosine phosphatase receptor type O (PTPRO) from breast cancer cells inactivated STAT3 and STAT6 in macrophages, polarizing macrophages into an M1-like phenotype and suppressing tumor cell invasion and migration 50. EVs from oral squamous cell carcinoma (OSCC) cells were found to mediate the transfer of THBS1 to macrophages and induce M1-like polarization by activating p38, Akt, and SAPK/JNK signaling 51.

### T-EV-lipids affect phenotypes of macrophages

Membrane lipids, especially arachidonic acid (AA), are essential for the fusion of EVs and plasma membranes 52, 53. Linton et al. found that EVs of the ascites-derived AsPC-1 cell line had a much larger proportion of phospholipid-esterified AA than EVs from other pancreatic ductal adenocarcinomas (PDAC) cell lines, which is the most noticeable difference between EVs from various PDAC cell lines 54. They reported that the AA component of phospholipids made it more likely for AsPC-1 EVs to fuse with macrophages 54. More importantly, their results suggested that AA was transported from AsPC-1 cells to macrophages via EVs and would cause a big increase in the production of PGE2 and pro-tumoral molecules (e.g., IL-1, IL-6, MCP-1, MMP-9, TNF, and VEGF), as well as polarize macrophages into an immunosuppressive M2-like phenotype 54.

## Tumor cell-derived EVs affect the immune functions of macrophages

It is commonly accepted that the immune system is comprised of two fundamental components: innate immunity and adaptive immunity. The innate immune system is the body's initial line of defense against pathogens (e.g., bacteria and viruses) and tumor cells 55. As the prime immune cells in innate immunity, macrophages engulf bacteria and tumor cells and secrete antimicrobial mediators, playing critical roles in bacterial recognition and elimination, as well as tumor clearance 55. Also, theoretically, the innate immune system's stimulation is required to develop adaptive immune responses against cancer 56.

By regulating macrophages, T-EVs participate in the innate immune response. Epidermal growth factor receptor positive (EGFR^+^) T-EVs could suppress the activation of IRF3 and type I interferon, thereby impairing the host's innate antiviral immunity 57. Moreover, T-EVs are effective in regulating macrophage immune functions. This is achieved through controlling macrophage activities such as cytokine production, expression of immune regulatory molecules on the membrane, phagocytosis and antigen presentation (Figure 2B-2E).

### Cytokine secretion

Cytokines have an important role as mediators of cell-to-cell communication in the TME. Cancer changes the synthesis and function of many cytokines which contribute to host anti-tumor responses 58. Macrophages secrete multiple cytokines that regulate tumor development and anti-tumor immunity. Some cytokines facilitate the polarization of macrophages to an anti-tumor state and promote inflammation to eliminate tumor cells, while others have the opposite effect 59-62. The cytokines that macrophages release also have other functions, like regulating angiogenesis and recruiting macrophages 63.

Certain T-EVs can stimulate the secretion or expression of pro-inflammatory cytokines (e.g., IFN-γ, IL-1β, and TNF-α), anti-inflammatory cytokines (e.g., Arg-1, TGF-β, and IL-10), chemokines (e.g., CCL1, CCL2, and MCP-1) and angiogenic factors (e.g., VEGF). Other T-EVs, however, inhibit the secretion of pro-inflammatory cytokines and co-stimulatory molecules (Figure 2B and Table 1).

T-EVs can affect inflammation via regulating the secretion of pro-inflammatory and anti-inflammatory factors by macrophages. Li et al. reported that the inhibition of lung cancer cell EV-miR101 promoted IL-1A and IL-6 expression by macrophages and led to inflammation in the TME by targeting CDK8 65. Ham et al. discovered that bone marrow-derived macrophages produced IL-6 in response to EV-glycoprotein 130 (gp130) of breast cancer cells, which in turn induced pro-survival macrophages through gp130/STAT3 signaling 67. Another study by Liang et al. suggested that EV-TRIM59 generated from lung cancer cells regulated ABHD5 proteasomal degradation to activate macrophage NLRP3 inflammasome signaling pathway, thereby promoting tumor progression via IL-1β production 64. Wei et al. demonstrated that the activation of macrophage NOD1 by CRC cell-derived EVs boosted the secretion of IL-6 and TNF-α, promoting CRC cell proliferation and migration 74. Of note, HCC-derived EV-lncRNA TUC339 could decrease the expression of pro-inflammatory cytokines as well as co-stimulatory molecules after being internalized by THP-1 cells 36.

Chemokines participate in the recruitment and education of macrophages and mediate cancer-related inflammation at the tumor site 75, 76. The release of chemokines by macrophages is also regulated by T-EVs. For instance, Gerloff et al. reported that melanoma EV delivered miR-125b-5p to macrophages and reinforced macrophage M1 activation by inducing CCL1, CCL2, and IL-1β production, hence promoting cancer-associated inflammation and myeloid cell recruitment 73.

The secretion of other cytokines, such as vascular endothelial growth factor C (VEGFC) and transforming growth factors (TGFs), is also influenced by T-EVs. Sun et al. demonstrated that EV-IRF-2 from CT26 cells induced the release of VEGFC by macrophages 69. Costa-Silva et al. found that uptake of PDAC-derived EVs by Kupffer cells induced TGF-β release and fibronectin generation of hepatic stellate cells 72.

### Expression of immune regulatory molecules on the membrane

Macrophage surface molecules, including antigens, receptors and other molecules mediate the immune responses of macrophages. Studies have shown that T-EVs regulate the levels of immune regulatory molecules on membrane of macrophages to affect immune suppression and disease progression (Figure 2C and Table 2).

The PD-L1/PD-1 signaling axis plays a critical role in tumor immunosuppression since it serves as an immune checkpoint and “don't eat me” signal. This axis inhibits T lymphocyte activation and increases the immunological tolerance of tumor cells, hence enabling tumor immune escape 91. In human and murine studies, macrophages in the TME displayed significant levels of functional PD-L1 expression 92. Several studies have proved that T-EVs foster tumor progression by increasing PD-1/PD-L1 levels in macrophages 80. According to research, intrahepatic cholangiocarcinoma cell-derived EV miR-183-5p upregulated PD-L1-expressing macrophages via the miR-183-5p/PTEN/AKT/PD-L1 pathway 80. Chen et al. revealed that GOLM1 increased PD-L1 expression in macrophages by enabling the transfer of PD-L1 to macrophages via EVs, hence increasing TAM infiltration in HCC 93. Yin et al. reported that HCC-EV therapy suppressed macrophage MHC-II expression while upregulating PD-L1 and CD80 83. As for PD-1, Wang et al. demonstrated that GC cells produced EVs to stimulate PD-1^+^ TAM production, which impaired CD8^+^ T-cell function 94.

CD39 functions as a “molecular switch” that regulates the inflammatory reactions of macrophages 95. Specifically, macrophages create immunosuppressive adenosine by hydrolyzing self-released ATP through CD39 96-98. Lu et al. found that EV-circTMEM181 sponged with miR-488-3p to upregulate macrophage CD39 level, which increased adenosine production and thus impaired anti-tumor immunity and caused anti-PD-1 resistance 88.

Furthermore, T-EVs may regulate the expression of a set of M1 (e.g., CD80, CD86, MHC-II, and TLR4) and M2 (e.g., CD163 and CD206) markers 27, 40, 83, 90 on membrane of macrophages. The altered expression levels of these molecules imply macrophage phenotype switch.

So far, a considerable amount of research has been conducted on PD-1/PD-L1 66, 80-82, 84, 94, while limited attention has been paid to other macrophage surface molecules, such as FasL and MET.

### Phagocytosis

Phagocytosis is a major characteristic of macrophages. Macrophages engulf and eliminate invading microorganisms and damaged cells, providing an effective immune response and maintaining homeostasis 99. According to research, T-EVs may either stimulate or inhibit macrophage-mediated phagocytosis (Figure 2D).

Macrophage-mediated antibody-dependent cellular phagocytosis (ADCP) is of great importance to tumor cell clearance. The stimulation of macrophage-mediated ADCP seems to be a significant mechanism behind the anti-tumor effects of a variety of therapeutic antibodies 99. Loss or mutation of TP53 is a key mediator of chemoresistance for various malignant tumors, including B-cell malignancies 100, 101. Izquierdo et al. revealed that loss of TP53 function in lymphoma led to increased EV production and that TP53-deficient lymphoma B-cell EVs inhibited Fc receptor-dependent macrophage ADCP and induced chemoimmunotherapy resistance through PD-L1 expression. Notably, neutralizing PD-L1 on the shTP53-EVs restored the anti-tumor potential of macrophages, as measured by ADCP 101. However, according to Su et al., macrophages undergone ADCP suppressed NK cell-mediated antibody-dependent cell-mediated cytotoxicity (ADCC) as well as T cell-mediated cytotoxicity, thereby having a detrimental effect on breast cancer immunosuppression 102. Furthermore, the combination of anti-HER2 antibodies with PD-L1 and IDO inhibitors improved anti-tumor immunity and anti-HER2 therapeutic effectiveness in murine models 102. Consequently, therapeutic antibodies combined with immune checkpoint blockade may have synergistic benefits in the treatment of cancer 102.

The phagocytic ability of macrophages is essential for the clearance of EVs from circulation and is stimulated by “eat me” signals (e.g., PS) and suppressed by “don't eat me” signals (e.g., CD47) 103-105. Yu et al. revealed that the higher level of CD47 and lower level of PS on T-EVs as compared to heterogeneous EVs might explain why T-EVs had a longer lifespan 105. In addition to CD47 and PS, there might be other “eat me” and “don't eat me” signals on T-EVs that can impact the ability of macrophages to phagocytose T-EVs, such as PD-L1, CD24, and CRT, and the interactions between those molecules have yet to be researched.

The phagocytic ability of macrophages could also be evaluated by the phagocytosis activity of the ovalbumin, yeast, or latex beads-rabbit IgG-FITC complex. Wang et al. evaluated phagocytosis levels of PD-1^+^ macrophages by analyzing the proportion of Ovalbumin^+^ macrophages. They discovered that gastric cancer cells significantly educated monocytes into PD-1^+^ macrophages by secreting EVs in vitro and in vivo, and PD-1^+^ macrophages phagocytosed significantly less ovalbumin in vitro than PD-1^-^ macrophages did 94. Moradi-Chaleshtori et al. revealed that the capacity of macrophages to phagocytose yeast was enhanced by 4T1 breast cancer cell-derived EVs containing miR-130 90. Kamerkar et al. found that after the uptake of HCC cell-derived EVs carrying up-regulated lncRNA TUC339, the phagocytosis of the latex beads-rabbit IgG-FITC complex was inhibited in THP-1 cells (commonly used model cells for macrophages), while the opposite effect was observed upon TUC339 suppression 36.

### Antigen presentation

Capture, endocytosis, and antigen presentation are crucial aspects of macrophage functions, which connect innate and adaptive immunity 106. Macrophages engulf pathogens, process their antigens, and present peptide fragments bound to human leukocyte antigen (HLA) molecules on their surface. This process alerts the immune system that pathogens are present 106. The levels of co-stimulatory and antigen-presenting molecules (e.g., MHC-I, MHC-II, CD80, and CD86) are significantly increased when macrophages are activated.

According to studies, T-EVs may facilitate macrophage antigen presentation (Figure 2E). The expression of murine immune region-associated (Ia) antigen on the surface of macrophages is crucial to their ability to present antigens 107. In the presence of Ia antigen plus a suitable “foreign” antigen, macrophages can induce the formation of T_H_ cells. The T_H_ cells may activate both cytotoxic T-lymphocytes and antibody-producing B-lymphocytes subsequently. Therefore, inhibiting Ia antigen expression on macrophages may limit both cellular and humoral immune responses. Taylor and Black discovered that plasma membrane-derived vesicles shed by metastatic variants of the murine B16 melanoma significantly inhibited the stimulation of macrophage Ia antigen expression, the earliest stage in the establishment of an immune response 108. Aspirin was capable of reversing membrane vesicle-induced inhibition, suggesting that this inhibition was caused by an increase in prostaglandin production 108. In addition, MHC-II, a key molecule in macrophage antigen presentation, is highly expressed in M1-polarized macrophages. Since T-EVs have the potential to induce M1 polarization, it can be hypothesized that T-EVs can affect the antigen-presenting function of macrophages by upregulating MHC-II expression 109.

## Perspectives

**Figure 3:**
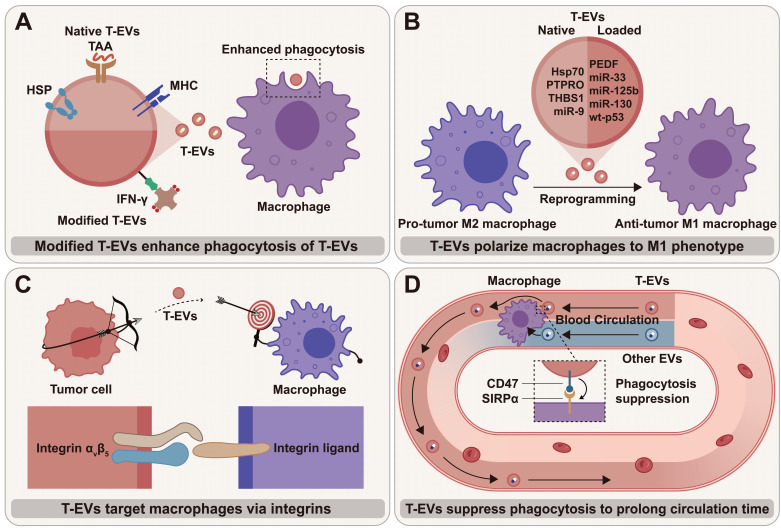
The therapeutic potential of macrophage-targeted T-EVs: (**A**) Using modified T-EVs as vaccines that stimulate macrophages to clear T-EVs; (**B**) T-EVs with native and loaded cargos polarize macrophages toward an anti-tumor M1 phenotype; (**C**) T-EVs can target macrophages via integrins and their ligands on the membrane; (**D**) The blood circulation time of T-EVs is likely to be longer than that of non-tumor-cell-derived EVs due to the higher amount of “don't eat me” signal CD47 and the lower level of “eat me” signal PS.

As important cellular mediators, T-EVs regulate macrophage phenotypes and functions by transporting various cargos to macrophages, thereby influencing tumor progression and cancer treatment. T-EVs that target and regulate macrophages are considered promising anti-tumor therapeutic targets, either alone or in combination therapies (Figure 3). A promising way to treat cancer is to explore T-EV's promoting potential and reverse their inhibitory effects on macrophage anti-tumor activities. T-EVs targeting macrophages can be also used for drug delivery, offering multiple advantageous outcomes.

### Exploring promoting potential of T-EVs on anti-tumor activities of macrophage

“Eat me” and “don't eat me” signals together control the process by which macrophages phagocytose tumor cells. Since CD47 can competitively occupy SIRPα, the overexpression of CD47 on T-EVs may disrupt CD47/SIRPα signal and lead to enhanced macrophage-mediated phagocytosis of tumor cells 110. Employing CD47-overexpressed T-EVs instead of anti-CD47 antibodies to block CD47/SIRPα signal has the advantage that T-EVs can carry therapeutics to kill tumor cells around macrophages, thus “killing two birds with one stone”. Thus, T-EVs with highly expressed “don't eat me” signals are promising delivery vehicles for cancer therapeutics.

T-EVs inherit tumor antigens from tumor cells, making them a source of tumor antigens that the immune system can identify. Therefore, T-EVs can be employed as tumor vaccines that stimulate immune responses against not only tumor cells but also T-EVs (Figure 3A). Shi et al. modified EVs of prostate cancer cells with interferon-γ (IFN-γ) and created a T-EV vaccine to help suppress tumor development 111. These biotinylated T-EVs contained tumor-associated antigens and immobilized streptavidin (SA)-tagged bioactive cytokines on the surface. They extended the lifespan of prostate cancer mice by increasing the quantity of M1 macrophages and enhancing the ability of M1 macrophages to engulf T-EVs 111. The use of T-EVs to activate the anti-tumor function and phenotype of macrophages is of great importance for tumor therapy, owing to the abundance 112 and phagocytic potential of macrophages. Although the immune response that T-EV vaccine induces is insufficient to kill tumor cells, T-EV vaccine shows adequate ability to stimulate the immune response to eliminate T-EVs, weakening T-EV-mediated immune escape. Thus, as shown in the study by Shi et al. 111, tumor vaccine and T-EV vaccine have a synergistic effect in inducing anti-tumor immunity.

### Reversing inhibiting effects of T-EVs on anti-tumor activities of macrophage

Generally, a viable method to treat cancer is to use small molecule inhibitors or antibodies that target specific signaling components to stop the inhibiting effects of T-EVs on macrophages. Given the pro-tumor properties of M2 macrophages, inhibiting macrophage M2 polarization and suppressing TAM infiltration with T-EVs that contain effector molecules can be promising therapeutic strategies against tumors (Figure 3B). For instance, Su et al. modified the cargos inside T-EVs by transfecting Panc-1 cells and successfully achieved stable expression of miR-155 and miR-125b2 in macrophages, which converted macrophages from M2 to M1 subtype 113. Jang et al. extracted EVs from epigallocatechin-3-gallate (EGCG)-treated 4T1 cells and discovered that miR-16 was increased in tumor cells and could be transmitted to TAMs through EVs, thereby suppressing TAM infiltration and M2 polarization 114.

### Employing macrophage-targeted T-EVs as drug delivery systems

T-EVs can target macrophages, and integrins are the molecular basis for T-EVs' targeting ability (Figure 3C). According to research, T-EVs express integrin α_v_β_5_ that specifically binds to Kupffer cells and contributes to liver tropism 115. Therefore, strategies targeting T-EV integrins may efficiently block the organ-tropic metastasis of tumors by cutting off the interactions between T-EVs and tissue-resident macrophages (TRMs). More importantly, the targeting ability of T-EVs makes it feasible to deliver therapeutics accurately to macrophages. The results of the studies so far suggest that T-EVs have considerable potential to deliver various therapeutic agents, including small molecule drugs and nucleic acids, to macrophages 116 (Table 3). The surface of T-EVs has been modified to improve transport efficiency and therapeutic effect, and the natural cargos of T-EVs have been discarded in some cases.

The macrophage-targeting ability of T-EVs could be enhanced when the “don't eat me” and “eat me” signals are smartly used. In a study by Liu et al., matrix metalloproteinase 2 (MMP2) was exploited to regulate PS externalization on the surface of the PEGylated nanoparticles (NPs) in order to achieve tumor-specific macrophage targeting 117. In non-tumor tissues, the surface-anchored PEG may impede phagocytosis, but in tumors, the increased MMP2 would externalize PS for TAM-targeted phagocytosis 117. Similar techniques may be used to boost the capacity of T-EVs to target macrophages.

Compared to EVs from other cells, T-EVs have a substantial advantage in blood circulation time (Figure 3D). Macrophages are the key to T-EV clearance from systemic circulation 120. As proven by Yu et al., the higher amount of CD47 and the lower level of PS on T-EVs compared to heterogeneous EVs likely result in a longer lifespan 105. The prolonged blood circulation time may lead to reduced toxicity to normal cells and stable drug release.

Despite this, it is still being determined whether native T-EVs can safely serve as drug carriers. The high level of immunosuppressive PD-L1 expression in native T-EVs raises the necessity to consider whether it may promote the progression of tumors 18. Because of this, using T-EVs for medication delivery requires further research to prevent or minimize potential adverse effects.

## Conclusions

Overall, this review summarizes the impact of T-EVs on macrophage phenotype and immune function, along with the underlying molecular mechanisms. More importantly, it presents several treatment strategies based on the regulation of T-EVs on macrophages, which are promising methods for treating cancer. Developing new strategies and combination regimens with enhanced specificity profiles is essential to overcome therapy resistance and eradicate cancer. Further research is needed to fully comprehend the complex interactions between T-EVs and macrophages and how these interactions influence cancer treatment.

## Supplementary Material

Supplementary table.

## Figures and Tables

**Table 1:** T-EVs regulate macrophage cytokine secretion.

EV source	Key molecules	Mode of action or pathways	Macrophage cell line	Cytokine secretion	Functions	Ref.
Lewis lung cancer cell	EGFR	Activating MEKK2	Peritoneal macrophage	IFN-β↓	Suppressing innate antiviral immunity	57
Lung cancer cell	TRIM59	Promoting ABHD5 proteasomal degradation and inducing NLRP3 inflammasome activation	THP-1	IL-1β↑	Promoting tumor progression	64
Hypoxic lung tumor cell	miR-101	CDK8	THP-1 & U937	IL-1A↑ IL-6↑	Inducing inflammation in the TME	65
Lewis lung carcinoma cell	/	/	Peritoneal macrophage	IL-6↑ IL-10↑	/	66
Breast cancer cell	/	gp130/STAT3	BMDM	IL-6↑	Establishing a pro-tumorigenic TME	67
Lung cancer cell	miR-21/miR-29a	TLR8/NF-κB	RAW 264.7	IL-6↑ TNF-α↑	Promoting tumor growth and dissemination	68
Colorectal cancer cell	IRF-2	/	RAW 264.7	VEGFC↑	Promoting tumor metastasis in sentinel lymph nodes	69
Breast cancer cell	miR-183-5p	PPP2CA/NF-κB	BMDM	IL-1β↑ IL-6↑ TNF-α↑	Promoting tumor progression	70
Breast cancer cell	Annexin II	p38MAPK/NF-κB/STAT3	Macrophage	IL-6↑ TNF-α↑	Promoting tumor metastasis to brain	71
Hepatocellular carcinoma cell	lncRNA TUC339	/	THP-1	IL-1β↓ TNF-α↓	/	36
Hypoxic ESCC cell	hsa-circ-0048117	miR-140/TLR4	THP-1	Arg-1↑ IL-10↑ TGF-β↑	Promoting tumor invasion and metastasis	40
Pancreatic cancer cell	AA	/	THP-1	IL-1β↑ IL-6↑ MCP-1↑ MMP-9↑ PGE2↑ TNF-α↑ VEGF↑	Promoting tumor progression	54
PDAC cell	MIF	/	Human Kupffer cell	TGF-β↑ Fibronectin↑	Promoting pre-metastatic niche formation and liver metastasis	72
Melanoma cell	miR-125b-5p	Lysosomal acid lipase A (LIPA)	THP-1	IL-1β↑ CCL1↑ CCL2↑	/	73

**Table 2:** T-EVs regulate the levels of immune regulatory molecules on membrane of macrophages.

EV source	Key molecules	Mode of action or pathways	Macrophage cell line	Changes in immune regulatory molecules	Functions	Ref.
Ovarian cancer cell	miR-155-5p	miR-155-5p/PD-L1	THP-1 & PBMC	PD-L1↓	Suppressing macrophage infiltration and tumor growth, promoting cytotoxic T cell activation	77
HNSCC cell	CD73	NF-κB	THP-1	LAG-3↑ PD-1↑ PD-L1↑	Promoting malignant progression	78
Colorectal cancer cell and multiple myeloma cell	/	TLR4/NF-κB	THP-1	PD-L1↑	Creating an immunosuppressive microenvironment	79
Lung cancer cell	HMGB-1	TLR2/NF-κB	PBMC & peritoneal macrophage	PD-L1↑	Promoting tumor metastasis	66
Intrahepatic cholangiocarcinoma cell	miR-183-5p	miR-183-5p/PTEN/AKT/PD-L1	Macrophage	PD-L1↑	Promoting immune suppression and tumor progression	80
ER-stressed hepatocellular carcinoma cell	miR-23a-3p	miR-23a/PTEN/AKT	THP-1	PD-L1↑	Inhibiting T-cell function	81
Colorectal cancer cell	miR-21-5p and miR-200a	PTEN/AKT and SCOS1/STAT1	THP-1 & RAW264.7	PD-L1↑	Suppressing CD8^+^ T cell activities	82
Hepatocellular carcinoma cell	miR-146a-5p	/	Peritoneal macrophage & BMDM & RAW264.7	MHC-II↓ PD-L1↑ CD80↑	Inhibiting T cell response	83
OSCC cell	CMTM6	/	THP-1	PD-L1↑	Promoting malignant progression	84
ER-stressed breast cancer cell	miR-27a-3p	MAGI2/PTEN/PI3K /AKT	THP-1	PD-L1↑	Inhibiting CD8^+^ T cells	85
H. pylori-infected gastric cancer cell	Mesenchymal-epithelial transition factor (MET)	/	THP-1 & PBMC	MET↑	Promoting tumor growth and progression	86
Hypoxia pre-challenged ESCC cell	hsa-circ-0048117	miR-140/TLR4	THP-1	TLR4↑	Promoting tumor metastasis	40
Follicular lymphoma cell	miR-7e-5p downregulation	/	Peritoneal macrophage	FasL↑	Inducing stromal M1 macrophage apoptosis	87
Hepatocellular carcinoma cell	circTMEM181	miR-488-3p/CD39	THP-1	CD39↑	Causing immunosuppression and anti-PD-1 resistance	88
Hepatocellular carcinoma cell	/	STAT3	THP-1 & RAW264.7	PD-L1↑	/	89
Melatonin-treated hepatocellular carcinoma cell	/	STAT3	THP-1 & RAW264.7	PD-L1↓	/	89
Colorectal cancer cell	miR-934	PTEN/PI3K/AKT	THP-1	CD163↑ CD206↑	Promoting liver metastasis	27
Breast cancer cell	Loaded miR-130	/	Peritoneal macrophage	CD86↑ CD206↓	Suppressing migration and invasion	90

**Table 3:** Macrophage-targeted T-EVs are employed as drug delivery systems.

Tumor cell line	Carrier type	Surface modification	Effector molecules	Effects	Ref.
H22	T-EVs	/	IONs	Promoting M1 polarization and inhibiting tumor growth	118
Panc-1	T-EVs	/	miR-155 and miR-125b	Re-programming macrophages from an M2 phenotype back to an M1 phenotype	113
CT26	Exosomes-thermosensitive liposomes hybrid nanovesicles	CD47 overexpression	Photothermal agent ICG and R837	Improving macrophage-mediated phagocytosis of tumor cells by blocking CD47 signal, promoting tumor-associated antigen generation	110
RM-1	T-EVs	IFN-γ modification	/	Increasing M1 macrophages quantity, promoting M1 macrophages to engulf RM-1 cell-derived EVs	111
4T1	Tumor cell apoptotic bodies	/	CpG	Polarizing macrophages to the M1 phenotype, enhancing macrophage antigen presentation and phagocytic ability	119
