# Supplementary material for: Tumor Cell-derived Extracellular Vesicles in Modulating Phenotypes and Immune Functions of Macrophages: Mechanisms and Therapeutic Applications
Source: J Cancer. 2023 May 8;14(8):1321–34. doi: 10.7150/jca.84632 (PMC10240675; doi:10.7150/jca.84632)
Supplement: Supplementary file 1 — Supplementary table. [file jcav14p1321s1.pdf]

Table S1. T-EVs regulate macrophage phenotypes.

Cargos	Donor cell	Regulation of cargos	Targets	Macrophage Cell Line	Biological function on macrophage	Biological function	Ref.
miR-9	HNSCC cell	HPV+	PPARδ	THP-1	M1 polarization	Increasing HPV+ HNSCC radiosensitivity	[1]
miR-15b	Hepatocellular carcinoma cell	Arsenite treatment	LATS1/Hippo	THP-1	M2 polarization	Promoting tumor cell proliferation, migration and invasion	[2]
miR-16	Multiple myeloma cell	/	IKKα/βcomplex	U937	M2 polarization	Supporting tumor cell growth	[3]
miR-19b-3p	Lung adenocarcinoma cell	/	PTPRD/STAT3	THP-1	M2 polarization	Facilitating tumor cell invasion, migration and tumor metastasis	[4]
miR-21	Head and neck cancer cell	Snai1 overexpression	PDCD4/IL-12A	PBMC	M2 polarization	Promoting tumor progression and indicating the real-time situation in the TME	[5]
miR-21	Bladder cancer cell	/	PTEN/p-AKT/STAT3/6	THP-1 & BMDM	M2 polarization	Promoting tumor cell growth, migration and invasion	[6]
miR-21-3p/miR-125b-5p/ miR-181d-5p	Epithelial ovarian cancer (EOC) cell	Hypoxic condition	SOCS4/5/STAT3	U937	M2 polarization	Promoting EOC cell proliferation and migration in a feedback loop	[7]
miR-21-5p	Esophageal squamous cell carcinoma cell	/	PTEN/PI3K/AKT/STAT6	THP-1	M2 polarization	Promoting tumor cell migration and invasion	[8]
miR-25-3p/miR-130b-3p/miR-425-5p	Colorectal cancer cell	CXCL12/CXCR4 activation	PTEN/PI3K/AKT	THP-1 & Raw 264.7	M2 polarization	Enhancing epithelial-mesenchymal transition (EMT) and liver metastasis	[9]
miR-27a-3p	Glioblastoma cell	Normoxic or hypoxic condition	EZH1/KDM3A/CTGF	THP-1	M2 polarization	Promoting tumor cell proliferation, migration and invasion	[10]
miR-29a-3p	Oral squamous cell carcinoma cell	/	SOCS1/p-STAT6	THP-1	M2 polarization	Promoting tumor cell proliferation and invasion	[11]
miR-33/miR-130	Breast cancer cell	Electroporation	/	PBMC & Peritoneal macrophage	M1 polarization	Inhibiting tumor progression	[12]

Cargos	Donor cell	Regulation of cargos	Targets	Macrophage Cell Line	Biological function on macrophage	Biological function	Ref.
miR-125b/wt-p53	Lung adenocarcinoma cell	Transfection	/	J774	M1 polarization	Supporting anti-tumor environment	[13]
miR-138-5p	Breast cancer cell	/	KDM6B	THP-1 & Raw 264.7	M2 polarization	Promoting lung metastasis	[14]
miR-145	Colorectal cancer cell	/	Histone deacetylase 11	THP-1 & NOMO-1	M2 polarization	Promoting tumor progression	[15]
miR-146a-5p	Hepatocellular carcinoma cell	SALL4	NF-κB and pro-inflammatory factors	THP-1 & Raw 264.7	M2 polarization	Promoting TME formation and tumor progression	[16]
miR-155-3p/IL-6	Glioma cell	Hypoxic condition	IL-6-pSTAT3-miR-155-3p-autophagy-pSTAT3 positive feedback loop	U937 & THP-1	M2 polarization	Advancing immunosuppressive microenvironment formation and promoting glioma progression	[17]
miR-222	Adriamycin-resistant breast cancer cell	/	PTEN/AKT	THP-1	M2 polarization	Stimulating tumor growth and pre-metastatic niche formation	[18]
miR-301a-3p	Pancreatic cancer cell	Hypoxic condition	PTEN/PI3K	THP-1	M2 polarization	Promoting tumor cell migration, invasion, and EMT	[19]
miR-301a-3p	Esophageal squamous cell carcinoma cell	/	PTEN/PI3K/AKT	THP-1	M2 polarization	Promoting angiogenesis	[20]
miR-770	Non-small cell lung cancer cell	/	MAP3K1	THP-1	Inhibiting M2 polarization	Inhibiting tumor growth	[21]
miR-934	Colorectal cancer cell	/	PTEN/PI3K/AKT	THP-1	M2 polarization	Promoting liver metastasis	[22]
miR-940	Ovarian cancer cell	Hypoxic condition	/	U937	M2 polarization	Promoting tumor cell proliferation and migration	[23]
miR-1246	Lung tumor cell	/	/	THP-1	M2 polarization	Promoting tumor growth and anti-immune suppression	[24]
miR-1246	Glioma cell	Hypoxic condition	TERF2IP STAT3/NF-κB	U937	M2 polarization	Facilitating immunosuppressive microenvironment formation	[25]
miR-3184-3p	Glioblastoma cell	/	RSAD2	THP-1 & PBMC	M2 polarization	Promoting tumor cell proliferation, invasion, and migration	[26]

Cargos	Donor cell	Regulation of cargos	Targets	Macrophage Cell Line	Biological function on macrophage	Biological function	Ref.
let-7a	Lung adenocarcinoma cell	Hypoxic condition	mTOR signaling pathway	THP-1	M2 polarization	Suppressing host immunity and enhancing tumor progression	[27]
let-7i-5p/miR-221-3p	Medulloblastoma cell	/	PPARγ	Raw 264.7	M2 polarization	Closely related to tumor progression	[28]
lncARSR	Renal cell carcinoma cell	/	STAT3	THP-1	M2 polarization	Promoting tumor progression	[29]
lncRNA HCG18	Gastric cancer cell	/	miR-875-3p/KLF4	THP-1	M2 polarization	Promoting tumor development	[30]
lncRNA HLA-F-AS1	Colorectal cancer cell	/	miR-375/PFN1	THP-1	M2 polarization	Promoting tumor metastasis	[31]
lncRNA HMMR-AS1	Hepatocellular carcinoma cell	Hypoxic condition	miR-174a/ARID3A	THP-1	M2 polarization	Promoting tumor cell proliferation and tumor growth	[32]
lncRNA PART1	Hepatocellular carcinoma cell	/	miR-372-3p/TLR4	THP-1	M2 polarization	Exerting oncogenic effect	[33]
lncRNA RPPH1	Colorectal cancer cell	/	TUBB3	/	M2 polarization	Promoting tumor cell metastasis and proliferation	[34]
lncRNA TUC339	Hepatocellular carcinoma cell	/	/	THP-1 & U937	M2 polarization	/	[35]
circ_0001142	Breast cancer cell	Endoplasmic reticulum stress	circ_0001142/miR-361-3p/PIK3CB	THP-1	M2 polarization	Promoting tumor growth and liver metastasis	[36]
circ_C20orf11	Ovarian cancer cell	/	/	THP-1	M2 polarization	Enhancing DDP resistance	[37]
circNEIL3	Glioblastoma multiforme cell	EWSR1	IGF2BP3/YAP1	THP-1	M2 polarization	Promoting glioma progression	[38]
circPVT1	Lung cancer cell	/	miR-124-3p/EZH2	THP-1	M2 polarization	Promoting tumor cell proliferation, invasion and migration	[39]
circSAFB2	Renal cell carcinoma cell	/	miR-620/JAK1/STAT3	THP-1	M2 polarization	Promoting tumor progression	[40]
hsa_circ_0074854	Hepatocellular carcinoma cell	/	HuR	THP-1	Inhibiting M2 polarization	Suppressing tumor cell migration and invasion	[41]
ANXA1	Pancreatic cancer cell	/	/	THP-1	M2 polarization	Promoting tumor progression and metastasis	[42]

Cargos	Donor cell	Regulation of cargos	Targets	Macrophage Cell Line	Biological function on macrophage	Biological function	Ref.
CMTM6	Oral squamous cell carcinoma cell	/	ERK1/2	THP-1	M2 polarization	Promoting malignant progression	[43]
CXCL14	Prostate cancer cell	/	NF-κB	THP-1	M2 polarization	Promoting tumor progression	[44]
gp130	Breast cancer cell	/	gp130/STAT3	BMDM	Pro-survival phenotype	Establishing a pro-tumorigenic TME	[45]
gp130	Diffuse large B-cell lymphoma cell	/	STAT3	THP-1	M2 polarization	Reconstructing the tumor-promoting microenvironment	[46]
HSP70	Breast cancer cell	Mild hyperthermic stress	iNOS/Arg1	RAW 264.7	Mixed M1 and M2 polarization	Inducing a pro-inflammatory response in macrophages	[47]
HSP90	Metastatic oral cancer cell	CDC37/HSP90α/ HSP90β	/	THP-1	M2 polarization	/	[48]
IL-32	Esophageal squamous cell carcinoma cell	/	FAK/STAT3	/	M2 polarization	Promoting lung metastasis	[49]
PD-L1	Oral squamous cell carcinoma cell	Endoplasmic reticulum stress	/	THP-1	M2 polarization	Promoting tumor progression	[50]
PD-L1	Melanoma cell	Sulfasalazine treatment	IRF4/Egr1	RAW264.7	M2 polarization	Inducing anti-PD-1/PD-L1 therapy resistance	[51]
PEDF	Breast cancer cell	/	/	/	M1 polarization	/	[52]
PTPRO	Breast tumor cell	/	STAT3/STAT6	THP-1	M1 polarization	Suppressing tumor cell invasion and migration	[53]
THBS1	Oral squamous cell carcinoma cell	/	p38/AKT/SAPK/JNK	THP-1	M1 polarization	Promoting malignant migration	[54]
TIE2	Cervical cancer cell	/	/	THP-1	M2 polarization	Promoting angiogenesis	[55]
TIM-3	Melanoma cell	/	/	THP-1	M2 polarization	Promoting tumor cell growth and metastasis	[56]
Arachidonic acid (AA)	Pancreatic cancer cell	/	/	THP-1	M2 polarization	Promoting tumor progression	[57]

References

1. Tong F, Mao X, Zhang S, Xie H, Yan B, Wang B, et al. HPV + HNSCC-derived exosomal miR-9 induces macrophage M1 polarization and increases tumor radiosensitivity. Cancer Lett. 2020; 478: 34-44.
2. Li J, Xue J, Ling M, Sun J, Xiao T, Dai X, et al. MicroRNA-15b in extracellular vesicles from arsenite-treated macrophages promotes the progression of hepatocellular carcinomas by blocking the LATS1-mediated Hippo pathway. Cancer Lett. 2021; 497: 137-53.
3. Khalife J, Ghose J, Martella M, Viola D, Rocci A, Troadec E, et al. MiR-16 regulates crosstalk in NF-kappaB tolerogenic inflammatory signaling between myeloma cells and bone marrow macrophages. JCI Insight. 2019; 4(21): e129348.
4. Chen J, Zhang K, Zhi Y, Wu Y, Chen B, Bai J, et al. Tumor-derived exosomal miR-19b-3p facilitates M2 macrophage polarization and exosomal LINC00273 secretion to promote lung adenocarcinoma metastasis via Hippo pathway. Clin Transl Med. 2021; 11: e478.
5. Hsieh CH, Tai SK, Yang MH. Snail-overexpressing Cancer Cells Promote M2-Like Polarization of Tumor-Associated Macrophages by Delivering MiR-21-Abundant Exosomes. Neoplasia. 2018; 20: 775-88.
6. Lin F, Yin HB, Li XY, Zhu GM, He WY, Gou X. Bladder cancer cell-secreted exosomal miR-21 activates the PI3K/AKT pathway in macrophages to promote cancer progression. Int J Oncol. 2020; 56: 151-64.
7. Chen X, Zhou J, Li X, Wang X, Lin Y, Wang X. Exosomes derived from hypoxic epithelial ovarian cancer cells deliver microRNAs to macrophages and elicit a tumor-promoted phenotype. Cancer Lett. 2018; 435: 80-91.
8. Song J, Yang P, Li X, Zhu X, Liu M, Duan X, et al. Esophageal Cancer-Derived Extracellular Vesicle miR-21-5p Contributes to EMT of ESCC Cells by Disorganizing Macrophage Polarization. Cancers (Basel). 2021; 13: 4122.
9. Wang D, Wang X, Si M, Yang J, Sun S, Wu H, et al. Exosome-encapsulated miRNAs contribute to CXCL12/CXCR4-induced liver metastasis of colorectal cancer by enhancing M2 polarization of macrophages. Cancer Lett. 2020; 474: 36-52.
10. Zhao G, Yu H, Ding L, Wang W, Wang H, Hu Y, et al. microRNA-27a-3p delivered by extracellular vesicles from glioblastoma cells induces M2 macrophage polarization via the EZH1/KDM3A/CTGF axis. Cell Death Discov. 2022; 8: 260.
11. Cai J, Qiao B, Gao N, Lin N, He W. Oral squamous cell carcinoma-derived exosomes promote M2 subtype macrophage polarization mediated by exosome-enclosed miR-29a-3p. Am J Physiol Cell Physiol. 2019; 316: C731-C40.
12. Moradi-Chaleshtori M, Bandehpour M, Soudi S, Mohammadi-Yeganeh S, Hashemi SM. In vitro and in vivo evaluation of anti-tumoral effect of M1 phenotype induction in macrophages by miR-130 and miR-33 containing exosomes. Cancer Immunol Immunother. 2021; 70: 1323-39.
13. Trivedi M, Talekar M, Shah P, Ouyang Q, Amiji M. Modification of tumor cell exosome content by transfection with wt-p53 and microRNA-125b expressing plasmid DNA and its effect on macrophage polarization. Oncogenesis. 2016; 5: e250.

14. Xun J, Du L, Gao R, Shen L, Wang D, Kang L, et al. Cancer-derived exosomal miR-138-5p modulates polarization of tumor-associated macrophages through inhibition of KDM6B. Theranostics. 2021; 11: 6847-59.
15. Shinohara H, Kuranaga Y, Kumazaki M, Sugito N, Yoshikawa Y, Takai T, et al. Regulated Polarization of Tumor-Associated Macrophages by miR-145 via Colorectal Cancer-Derived Extracellular Vesicles. J Immunol. 2017; 199: 1505-15.
16. Yin C, Han Q, Xu D, Zheng B, Zhao X, Zhang J. SALL4-mediated upregulation of exosomal miR-146a-5p drives T-cell exhaustion by M2 tumor-associated macrophages in HCC. Oncoimmunology. 2019; 8: 1601479.
17. Xu J, Zhang J, Zhang Z, Gao Z, Qi Y, Qiu W, et al. Hypoxic glioma-derived exosomes promote M2-like macrophage polarization by enhancing autophagy induction. Cell Death Dis. 2021; 12: 373.
18. Chen WX, Wang DD, Zhu B, Zhu YZ, Zheng L, Feng ZQ, et al. Exosomal miR-222 from adriamycin-resistant MCF-7 breast cancer cells promote macrophages M2 polarization via PTEN/Akt to induce tumor progression. Aging (Albany NY). 2021; 13: 10415-30.
19. Wang X, Luo G, Zhang K, Cao J, Huang C, Jiang T, et al. Hypoxic Tumor-Derived Exosomal miR-301a Mediates M2 Macrophage Polarization via PTEN/PI3Kgamma to Promote Pancreatic Cancer Metastasis. Cancer Res. 2018; 78: 4586-98.
20. Shou Y, Wang X, Chen C, Liang Y, Yang C, Xiao Q, et al. Exosomal miR-301a-3p from esophageal squamous cell carcinoma cells promotes angiogenesis by inducing M2 polarization of macrophages via the PTEN/PI3K/AKT signaling pathway. Cancer Cell Int. 2022; 22: 153.
21. Liu J, Luo R, Wang J, Luan X, Wu D, Chen H, et al. Tumor Cell-Derived Exosomal miR-770 Inhibits M2 Macrophage Polarization via Targeting MAP3K1 to Inhibit the Invasion of Non-small Cell Lung Cancer Cells. Front Cell Dev Biol. 2021; 9: 679658.
22. Zhao S, Mi Y, Guan B, Zheng B, Wei P, Gu Y, et al. Tumor-derived exosomal miR-934 induces macrophage M2 polarization to promote liver metastasis of colorectal cancer. J Hematol Oncol. 2020; 13: 156.
23. Chen X, Ying X, Wang X, Wu X, Zhu Q, Wang X. Exosomes derived from hypoxic epithelial ovarian cancer deliver microRNA-940 to induce macrophage M2 polarization. Oncol Rep. 2017; 38: 522-8.
24. Pritchard A, Tousif S, Wang Y, Hough K, Khan S, Strenkowski J, et al. Lung Tumor Cell-Derived Exosomes Promote M2 Macrophage Polarization. Cells. 2020; 9: 1303.
25. Qian M, Wang S, Guo X, Wang J, Zhang Z, Qiu W, et al. Hypoxic glioma-derived exosomes deliver microRNA-1246 to induce M2 macrophage polarization by targeting TERF2IP via the STAT3 and NF-kappaB pathways. Oncogene. 2020; 39: 428-42.
26. Xu H, Li M, Pan Z, Zhang Z, Gao Z, Zhao R, et al. miR-3184-3p enriched in cerebrospinal fluid exosomes contributes to progression of glioma and promotes M2-like macrophage polarization. Cancer Sci. 2022; 113: 2668-80.
27. Park JE, Dutta B, Tse SW, Gupta N, Tan CF, Low JK, et al. Hypoxia-induced tumor exosomes promote M2-like macrophage polarization of infiltrating myeloid cells and microRNA-mediated metabolic shift. Oncogene. 2019; 38: 5158-73.
28. Zhu L, Yang Y, Li H, Xu L, You H, Liu Y, et al. Exosomal microRNAs induce tumor-associated macrophages via PPARgamma during tumor progression in SHH medulloblastoma. Cancer Lett. 2022; 535: 215630.

29. Zhang W, Zheng X, Yu Y, Zheng L, Lan J, Wu Y, et al. Renal cell carcinoma-derived exosomes deliver lncARSR to induce macrophage polarization and promote tumor progression via STAT3 pathway. Int J Biol Sci. 2022; 18: 3209-22.
30. Xin L, Wu Y, Liu C, Zeng F, Wang JL, Wu DZ, et al. Exosome-mediated transfer of lncRNA HCG18 promotes M2 macrophage polarization in gastric cancer. Mol Immunol. 2021; 140: 196-205.
31. Zhang J, Li S, Zhang X, Li C, Zhang J, Zhou W. LncRNA HLA-F-AS1 promotes colorectal cancer metastasis by inducing PFN1 in colorectal cancer-derived extracellular vesicles and mediating macrophage polarization. Cancer Gene Ther. 2021; 28: 1269-84.
32. Wang X, Zhou Y, Dong K, Zhang H, Gong J, Wang S. Exosomal lncRNA HMMR-AS1 mediates macrophage polarization through miR-147a/ARID3A axis under hypoxia and affects the progression of hepatocellular carcinoma. Environ Toxicol. 2022; 37: 1357-72.
33. Zhou J, Che J, Xu L, Yang W, Zhou W, Zhou C. Tumor-derived extracellular vesicles containing long noncoding RNA PART1 exert oncogenic effect in hepatocellular carcinoma by polarizing macrophages into M2. Dig Liver Dis. 2022; 54: 543-53.
34. Liang ZX, Liu HS, Wang FW, Xiong L, Zhou C, Hu T, et al. LncRNA RPPH1 promotes colorectal cancer metastasis by interacting with TUBB3 and by promoting exosomes-mediated macrophage M2 polarization. Cell Death Dis. 2019; 10: 829.
35. Li X, Lei Y, Wu M, Li N. Regulation of Macrophage Activation and Polarization by HCC-Derived Exosomal lncRNA TUC339. Int J Mol Sci. 2018; 19: 2958.
36. Lu C, Shi W, Hu W, Zhao Y, Zhao X, Dong F, et al. Endoplasmic reticulum stress promotes breast cancer cells to release exosomes circ_0001142 and induces M2 polarization of macrophages to regulate tumor progression. Pharmacol Res. 2022; 177: 106098.
37. Yin J, Huang HY, Long Y, Ma Y, Kamalibaike M, Dawuti R, et al. circ_C20orf11 enhances DDP resistance by inhibiting miR-527/YWHAZ through the promotion of extracellular vesicle-mediated macrophage M2 polarization in ovarian cancer. Cancer Biol Ther. 2021; 22: 440-54.
38. Pan Z, Zhao R, Li B, Qi Y, Qiu W, Guo Q, et al. EWSR1-induced circNEIL3 promotes glioma progression and exosome-mediated macrophage immunosuppressive polarization via stabilizing IGF2BP3. Mol Cancer. 2022; 21: 16.
39. Liu Y, Li L, Song X. Exosomal circPVT1 derived from lung cancer promotes the progression of lung cancer by targeting miR-124-3p/EZH2 axis and regulating macrophage polarization. Cell Cycle. 2022; 21: 514-30.
40. Huang X, Wang J, Guan J, Zheng Z, Hao J, Sheng Z, et al. Exosomal Circsafb2 Reshaping Tumor Environment to Promote Renal Cell Carcinoma Progression by Mediating M2 Macrophage Polarization. Front Oncol. 2022; 12: 808888.
41. Wang Y, Gao R, Li J, Tang S, Li S, Tong Q, et al. Downregulation of hsa_circ_0074854 Suppresses the Migration and Invasion in Hepatocellular Carcinoma via Interacting with HuR and via Suppressing Exosomes-Mediated Macrophage M2 Polarization. Int J Nanomedicine. 2021; 16: 2803-18.
42. Novizio N, Belvedere R, Pessolano E, Morello S, Tosco A, Campiglia P, et al. ANXA1 Contained in EVs Regulates Macrophage Polarization in Tumor Microenvironment and Promotes Pancreatic Cancer Progression and Metastasis. Int J Mol Sci. 2021; 22: 11018.

43. Pang X, Wang SS, Zhang M, Jiang J, Fan HY, Wu JS, et al. OSCC cell-secreted exosomal CMTM6 induced M2-like macrophages polarization via ERK1/2 signaling pathway. Cancer Immunol Immunother. 2021; 70: 1015-29.
44. Tian HY, Liang Q, Shi Z, Zhao H. Exosomal CXCL14 Contributes to M2 Macrophage Polarization through NF-kappaB Signaling in Prostate Cancer. Oxid Med Cell Longev. 2022; 2022: 7616696.
45. Ham S, Lima LG, Chai EPZ, Muller A, Lobb RJ, Krumeich S, et al. Breast Cancer-Derived Exosomes Alter Macrophage Polarization via gp130/STAT3 Signaling. Front Immunol. 2018; 9: 871.
46. Ling HY, Yang Z, Wang PJ, Sun Y, Ju SG, Li J, et al. Diffuse large B-cell lymphoma-derived exosomes push macrophage polarization toward M2 phenotype via GP130/STAT3 signaling pathway. Chem Biol Interact. 2022; 352: 109779.
47. Sen K, Sheppe AEF, Singh I, Hui WW, Edelmann MJ, Rinaldi C. Exosomes released by breast cancer cells under mild hyperthermic stress possess immunogenic potential and modulate polarization in vitro in macrophages. Int J Hyperthermia. 2020; 37: 696-710.
48. Ono K, Sogawa C, Kawai H, Tran MT, Taha EA, Lu Y, et al. Triple knockdown of CDC37, HSP90-alpha and HSP90-beta diminishes extracellular vesicles-driven malignancy events and macrophage M2 polarization in oral cancer. J Extracell Vesicles. 2020; 9: 1769373.
49. Sun Y, Qian Y, Chen C, Wang H, Zhou X, Zhai W, et al. Extracellular vesicle IL-32 promotes the M2 macrophage polarization and metastasis of esophageal squamous cell carcinoma via FAK/STAT3 pathway. J Exp Clin Cancer Res. 2022; 41: 145.
50. Yuan Y, Jiao P, Wang Z, Chen M, Du H, Xu L, et al. Endoplasmic reticulum stress promotes the release of exosomal PD-L1 from head and neck cancer cells and facilitates M2 macrophage polarization. Cell Commun Signal. 2022; 20: 12.
51. Liu N, Zhang J, Yin M, Liu H, Zhang X, Li J, et al. Inhibition of xCT suppresses the efficacy of anti-PD-1/L1 melanoma treatment through exosomal PD-L1-induced macrophage M2 polarization. Mol Ther. 2021; 29: 2321-34.
52. Moradi-Chaleshtori M, Koochaki A, Shojaei S, Paryan M, Safarzadeh M, Hashemi SM, et al. Overexpression of pigment epithelium-derived factor in breast cancer cell-derived exosomes induces M1 polarization in macrophages. Immunol Lett. 2022; 248: 31-6.
53. Dong H, Xie C, Jiang Y, Li K, Lin Y, Pang X, et al. Tumor-Derived Exosomal Protein Tyrosine Phosphatase Receptor Type O Polarizes Macrophage to Suppress Breast Tumor Cell Invasion and Migration. Front Cell Dev Biol. 2021; 9: 703537.
54. Xiao M, Zhang J, Chen W, Chen W. M1-like tumor-associated macrophages activated by exosome-transferred THBS1 promote malignant migration in oral squamous cell carcinoma. J Exp Clin Cancer Res. 2018; 37: 143.
55. Du S, Qian J, Tan S, Li W, Liu P, Zhao J, et al. Tumor cell-derived exosomes deliver TIE2 protein to macrophages to promote angiogenesis in cervical cancer. Cancer Lett. 2022; 529: 168-79.
56. Li X, Liu Y, Yang L, Jiang Y, Qian Q. TIM-3 shuttled by MV3 cells-secreted exosomes inhibits $CD4^+$ T cell immune function and induces macrophage M2 polarization to promote the growth and metastasis of melanoma cells. Transl Oncol. 2022; 18: 101334.
57. Linton SS, Abraham T, Liao J, Clawson GA, Butler PJ, Fox T, et al. Tumor-promoting effects of pancreatic cancer cell exosomes on THP-1-derived macrophages. PLoS One. 2018; 13: e0206759.
